# Comparison of the Efficacy of Cisplatin/Paclitaxel Versus Carboplatin/Paclitaxel in Improving Survival and Quality of Life in the Advanced Ovarian Cancer Patient Population: A Systematic Review and Meta-Analysis of Randomized Control Trials

**DOI:** 10.7759/cureus.51011

**Published:** 2023-12-23

**Authors:** Marium Mansoor, Firzah Shakil, Urba Jalal, Fatimah Shahid, Maira Jamal, Alishba S Ali, Fatima A Abbasi, Hamna Hijazi, Hamza Imran, Sapna Hirani, Aima Javaid, Ahshum Abu Bakar, Abdul Ahad Shah, Giustino Varrassi, Mahima Khatri, Satesh Kumar

**Affiliations:** 1 Medicine and Surgery, Allama Iqbal Medical College, Lahore, PAK; 2 Allergy and Immunology, Dow University of Health Sciences, Civil Hospital Karachi, Karachi, PAK; 3 Medical School, Allama Iqbal Medical College, Lahore, PAK; 4 Medical School, Rawalpindi Medical University, Rawalpindi, PAK; 5 Pediatrics, Hamdard College of Medicine and Dentistry, Karachi, PAK; 6 Cardiology, Shifa International Hospital Islamabad, Islamabad, PAK; 7 Medical School, Khyber Medical College, Peshawar, PAK; 8 Medical School, Dow University of Health Sciences, Civil Hospital Karachi, Karachi, PAK; 9 Medical School, Peoples University of Medical and Health Science, Nawabshah, PAK; 10 Medical School, Fatimah Jinnah Medical University, Lahore, PAK; 11 Medical School, Shalamar Medical and Dental College, Lahore, PAK; 12 Pain Medicine, Paolo Procacci Foundation, Rome, ITA; 13 Medicine and Surgery, Dow University of Health Sciences, Civil Hospital Karachi, Karachi, PAK; 14 Medicine and Surgery, Shaheed Mohtarma Benazir Bhutto Medical College, Karachi, PAK

**Keywords:** meta-analysis, advanced ovarian cancer, paclitaxel, carboplatin, cisplatin

## Abstract

Ovarian cancer, being one of the prevalent gynecological cancers, warrants a therapy that’s both effective and well tolerated. After extensive drug testing, combination regimens with paclitaxel plus platinum-based agents such as cisplatin/carboplatin and taxanes, have shown promising results for advanced ovarian cancer. We conducted a systematic review and meta-analysis of randomized controlled trials (RCTs) to compare the efficacy of two treatment regimens for advanced ovarian cancer: cisplatin/paclitaxel and carboplatin/paclitaxel.

PubMed (Medline), Science Direct, and Cochrane Library were searched from inception to March 2023. The meta-analysis included patients with histologically verified International Federation of Gynaecology and Obstetrics (FIGO) stages IIB to IV ovarian carcinoma who received either carboplatin/paclitaxel or cisplatin/paclitaxel. The primary outcomes were progression-free survival (PFS), overall survival (OS), quality of life (QOL), complete response rate (CRR), and partial response rate (PRR). The revised Cochrane Risk of Bias Tool 2.0 was used to assess the quality of the RCTs

The five RCTs chosen for this statistical analysis consisted of a total of 2239 participants, with 1109 receiving paclitaxel/cisplatin for treatment and the remaining 1130 receiving carboplatin/paclitaxel. Among all included outcomes, these reported significant findings: QoL (p-value=0.0002), thrombocytopenia (p=<0.00001), neurological toxicity (p-value=0.003), nausea/vomiting (p-value=<0.00001), myalgia/arthralgia (p-value=0.02), and febrile neutropenia (p-value=0.01).

We concluded that the carboplatin/paclitaxel doublet endows a better quality of life (QOL) to patients along with significantly fewer gastrointestinal and neurological toxicities when compared with the cisplatin/paclitaxel combination. However, the myelosuppressive effects of carboplatin/paclitaxel remain a point of concern and may require clinical management.

## Introduction and background

Ovarian carcinomas account for a staggering 23% of female reproductive tract cancers. Consequently, these tumors are ranked as the third-most prevalent neoplastic lesions in the field of gynecology, with the first and second being uterine and cervical carcinomas, respectively [1]. Among women, ovarian carcinoma is responsible for 47% of all cancer-related deaths. This elevated mortality rate can be attributed to the non-specific nature of early-stage presenting signs, coupled with the absence of reliable screening tools. In light of this, the diagnosis is often delayed and treatment is initiated when the cancer has progressed to an advanced stage [2-4]. Due to the increased disease prevalence and pathophysiological complications, there is a pressing need for treatment that is both effective and well-tolerated, given the significant physical and physiological challenges posed by disease progression [5].

Initially, alkylating agents and non-platinum-based regimens were optimized as the standard of care for epithelial ovarian cancer management. However, their markedly low expected response rates of 40 to 50% and low median survivals led to trials testing taxanes and platinum-based agents as possible alternatives [6]. Nejit et al. compared a four-drug regimen of a combination of hexamethylmelamine, cyclophosphamide, methotrexate, and 5-fluorouracil (hexa-CAF) composed mainly of alkylating agents with CHAP-5, another four-drug combination of alkylating agents, and a platinum analog cisplatin in an attempt to assess whether the inclusion of cisplatin in the regimen would affect patient outcomes. The trial concluded that the inclusion of cisplatin alleviated the treatment response rate, progression-free survival time, and overall survival significantly; therefore, establishing the superiority of platinum-agent-based combination therapy over non-platinum-based regimens [7,8]. Subsequently, multiple trials were conducted to further investigate the safety and efficacy of platinum-based combination chemotherapy regimens. Among the available treatment modalities, the cisplatin/paclitaxel doublet stood out in terms of both patient outcomes and treatment efficacy. Thereby, the platinum/taxane combination garnered recognition as the preferred first-line chemotherapeutic regimen for ovarian cancer within the realm of clinical oncology [9,10].

Cisplatin, despite its clinical efficacy, exhibited significant dose-limiting neurotoxicity, nephrotoxicity, and gastrointestinal toxicity [11]. Hence, the search for a better alternative remained underway until carboplatin, a relatively less toxic platinum analog, was taken up for testing and was found to demonstrate a better drug tolerability profile in comparison to its parent compound cisplatin [12]. However, the dose-limiting myelosuppression that accompanied carboplatin administration was a growing point of concern for researchers [13].

This prompted a number of randomized control trials (RCTs) that sought to ascertain which one of these two pharmacological agents would contribute to better patient outcomes in conjunction with paclitaxel for the treatment of advanced ovarian cancer. The primary objective of this meta-analysis is to comprehensively analyze the findings of five such RCTs that compare two key adjuvant chemotherapy protocols: cisplatin/paclitaxel against carboplatin/paclitaxel with respect to efficacy, drug toxicity, and quality of life.

## Review

Methods

Search Strategy and Data Sources

This meta-analysis was performed in accordance with the Preferred Reporting Items for Systematic Reviews and Meta-Analyses (PRISMA) guidelines statement [14].

A comprehensive electronic search was performed on three databases namely PubMed (Medline), Science Direct, and Cochrane Library, and no filters were applied to the study design. The following search strategy was used as the primary string; (("Cisplatin/paclitaxel") OR ("Cisplatin-paclitaxel") AND ("carboplatin/paclitaxel") OR ("carboplatin-paclitaxel") AND ("ovarian cancer") OR ("ovary cancer")) and we included relevant RCTs published from inception till March 20, 2023. Information about the search methodology is provided in Table 1. 

**Table 1:** Detailed search strategy

Database	Search strategy	Results
PubMed	(("Cisplatin/paclitaxel") OR ("Cisplatin-paclitaxel") AND ("carboplatin/paclitaxel") OR ("carboplatin-paclitaxel") AND ("ovarian cancer") OR ("ovary cancer"))	751
Science Direct	(("Cisplatin/paclitaxel") OR ("Cisplatin-paclitaxel") AND ("carboplatin/paclitaxel") OR ("carboplatin-paclitaxel") AND ("ovarian cancer") OR ("ovary cancer"))	699
Cochrane Library	(("Cisplatin/paclitaxel") OR ("Cisplatin-paclitaxel") AND ("carboplatin/paclitaxel") OR ("carboplatin-paclitaxel") AND ("ovarian cancer") OR ("ovary cancer"))	2624

Eligibility Criteria

The inclusion criteria used for the comprehensive search included recruitment of RCTs (in English) which reported patients with histologically verified International Federation of Gynaecology and Obstetrics (FIGO) stages IIB to IV ovarian carcinoma who had not undergone chemotherapy before and were divided randomly into two groups, with one group receiving cisplatin-paclitaxel as intervention while the other group was being given carboplatin-paclitaxel as the control. The RCTs were required to report at least one of the primary outcomes namely median progression-free survival time (PFS), overall survival (OS), quality of life (QoL), complete clinical response (CRR), and partial response rate (PRR). The secondary outcomes were the toxicities that included anemia, thrombocytopenia, neutropenia, febrile neutropenia, leukopenia, neuropathy, constipation, diarrhea, mucositis or stomatitis, nausea or vomiting, myalgia or arthralgia, alopecia, hypersensitivity or allergy, ototoxicity, dyspnea, nephrotoxicity, cardiotoxicity, and edema. 

We excluded studies that are non-English, systematic reviews, meta-analyses, narrative reviews, observational studies, cohort studies, case reports/ series, editorials, study protocols, abstracts, commentaries, letters, and the studies that document the outcomes for only one drug for example: outlining the efficacy for paclitaxel and discussing its outcomes without drawing comparisons in combination with cisplatin or carboplatin. The screening of the incorporated studies was in close association with pre-specified eligibility criteria and outcome measures.

Data Extraction and Quality Assessment

The articles that were obtained through the electronic search were exported into the EndNote Reference Library software so that any duplicates could be removed. Each study that satisfied the criteria to be pooled into this meta-analysis was reviewed by two independent researchers (MM and UJ) who performed a full-text review after the studies were initially screened on the grounds of titles and abstract reading. Any discrepancy was resolved after consulting a third independent researcher (SAS). The continuous outcome variables studied were defined as median PFS, OS, and QoL, whereas the dichotomous outcome variables included complete/partial clinical response and toxicities/adverse events all of which have been mentioned above. The two reviewers then extracted data on the following: (1) the study details (the author name, study design, year of study, and phase, the inclusion and exclusion criteria as well as the follow-up period); (2) the baseline characteristics of the patients such as the age, grading, staging, and size of the tumor, the performance, and the number of treatment cycles received.

To assess which of the PT arm or TC arm was better, we extracted the means and standard deviations for the following continuous outcomes: PFS, QoL, and OS. If any study reported the values in IQR, we calculated the SD using the following equation: SD= Change in IQR/1.35 [15].

And if any study reported more than one mean (SD) for the baseline and follow-ups, we calculated the mean change by simply subtraction (follow-up mean- baseline mean), to calculate the change in standard deviation, we used the following equation: SD change = √(〖SD(baseline)〗^2+〖SD(final)〗^2-2^r^SD(baseline)^SD(final)), where “r” is the correlation coefficient for the correlation between the baseline and final value. The baseline characteristics and the outcome data were all extracted on an online Microsoft Excel sheet. 

Two researchers (FS and MJ) assessed the quality of the RCTs included by using the revised Cochrane Risk of Bias tool (RoB) independently [16]. Allocation of treatment concealed from patients and investigators, selective reporting, blinding of outcome assessment, inadequate results, random sequence generation, and randomization of participants to the treatment are the seven areas for the judgment of the risk of bias. ‘Low risk’, ‘High risk’, and ‘uncertain’ are the ratings for these seven domains of the risk of bias. 

Statistical Analyses

The Review Manager 5.4 (Cochrane Collaboration, 2020) tool was used to perform the statistical analysis. For the continuous outcomes provided as raw data, mean values and standard deviations were retrieved. Continuous outcomes given in terms of effect sizes were analyzed via weighted mean differences (WMD) with 95% confidence intervals. The generic-inverse variance model was employed to design forest plots to calculate the mixed data for the continuous outcomes. For dichotomous outcomes, risk ratios (RR) along with their 95% confidence intervals were pooled after performing analysis using the random effects model. Throughout the analysis, a P-value of less than 0.05 was regarded as significant.

For each result, heterogeneity was calculated using the Higgins I^2 statistics and expressed as a percentage [17]. Low heterogeneity was indicated for I^2 values less than 50%, moderate heterogeneity was taken into account for I^2 values less than 75%, and high heterogeneity was evaluated for I^2 values greater than 75%. A greater than 75% I^2 value was subjected to sensitivity analysis.

Publication Bias

The funnel plots for the primary outcomes were simultaneously made for each outcome using the random effects model on the Review Manager 5.4 tool. 

Results

Study Selection

**Figure 1:**
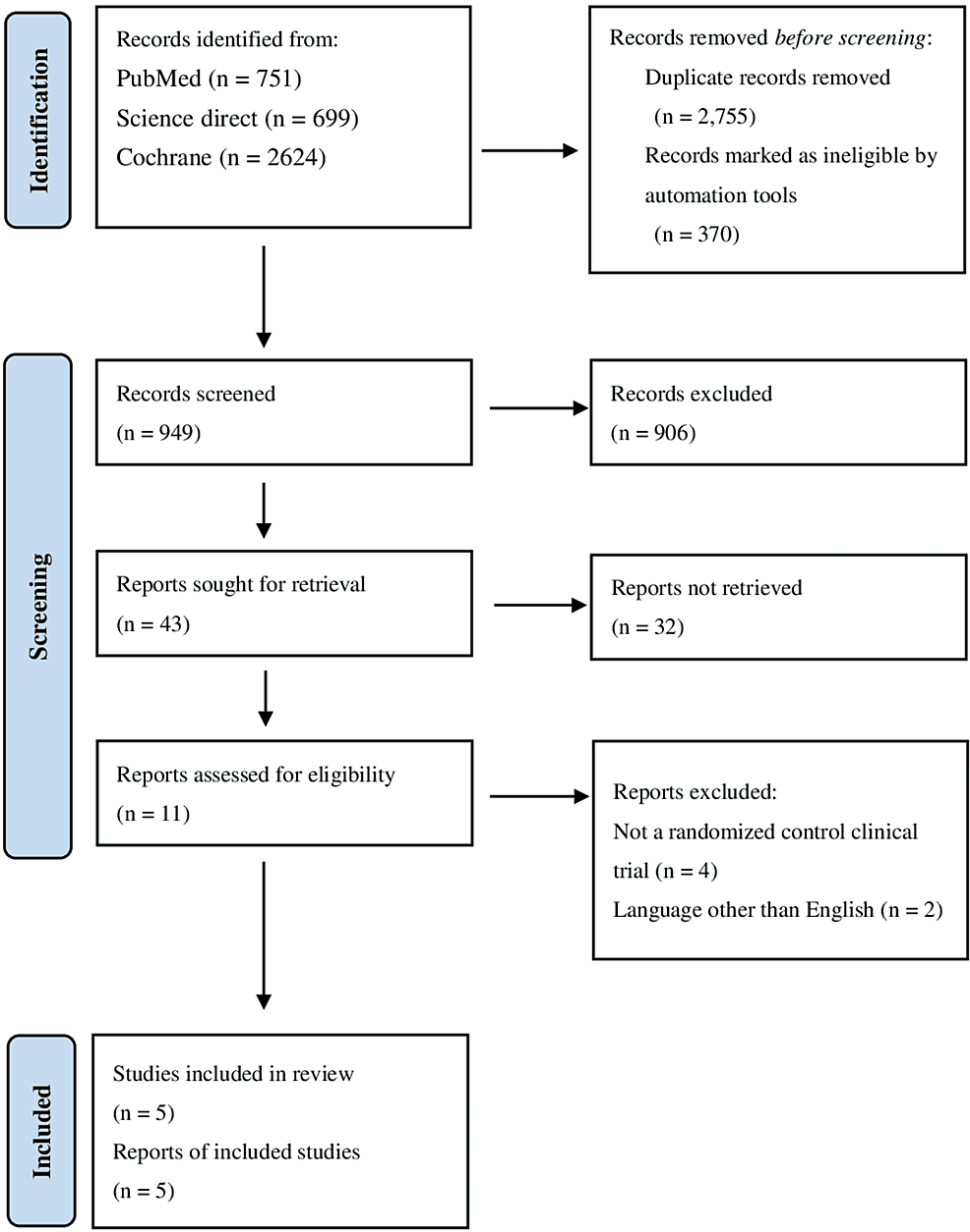
PRISMA flow diagram showing the study selection process

An extensive search of three databases yielded a total of 4.074 results. Around 949 records were screened, of which 906 articles were excluded after reviewing their titles and abstracts. The rest of the records were given a full-text review. In the end, five RCTs were chosen for the meta-analysis [18-22] (Figure 1).

Patients’ Demographics 

In the five RCTs included for the meta-analysis, there were a total of 2239 participants, among which 1109 received the paclitaxel/cisplatin drug group (intervention arm) for treatment and the remaining 1130 received the paclitaxel/carboplatin combination (control arm). Study characteristics for the included RCTs such as inclusion and exclusion criteria, follow-ups, and treatment dosages have been included as well (Table 2). Baseline patient characteristics namely tumor grading, tumor staging, number of treatment cycles received as well as ECOG performance have also been reported (Table 3).

**Table 2:** Study characteristics of included RCTs TC: paclitaxel and carboplatin, PT: paclitaxel and cisplatin, ECOG: Eastern Cooperative Oncology Group: FIGO: International Federation of Gynecology and Obstetrics, WHO: World Health Organization, GOG: Gynecologic Oncology Group, ETOC: epithelial tubo-ovarian cancer, PPSC: primary peritoneal serous carcinoma, AUC: area under the curve, PCS: primary cytoreductive surgery, GFR: glomerular filtration rate

S. No.	First Author	Year of Publication	Total Patients	Inclusion Criteria	Exclusion Criteria	Follow up	Treatment Doses	
							TC arm	PT arm
1	Bois et al. [18]	2003	783	(1) Patients with Histologically verified FIGO stages IIB–IV ovarian cancer; (2) Patients who underwent radical debulking surgery within 6 weeks of random assignment; (3) Minimum age for inclusion was 18 years; (4) Absolute neutrophil count (ANC) of at least 1.5 × 109 cells/L, platelet count of at least 100 × 109 cells/L, serum creatinine and bilirubin of no more than 1.25 × upper normal limit.	(1) Patients with ovarian tumors with low malignant potential; (2) an ECOG performance status of more than 2 or a Karnofsky index of less than 60%; (3) an estimated GFR of less than 60 mL/minute; (4) other malignancies; (5) previous chemotherapy, radiotherapy, or immunotherapy for ovarian cancer; (6) severe neuropathy or cardiopathy (arrhythmias and failure)	60 months	Paclitaxel at a dose of 185mg/m2 administered intravenously over 3 hours, followed by carboplatin (AUC 6) administered intravenously over 30-60 minutes	Paclitaxel was administered intravenously over 3 hours at a dose of 185mg/m2 followed by cisplatin administered intravenously over 30 minutes at a dose of 75mg/m2
2	Huang et al. [20]	2020	40	(1) Patients were recruited between January 2010 and December 2016 who had histologically confirmed FIGO stage IIIC high-grade serous-type ETOC and PPSC; (2) An initial PCS, a total of six cycles of weekly paclitaxel [80 mg/m2] plus either triweekly cisplatin [20 mg/m2] or triweekly carboplatin [AUC 5]	(1) Patients with other newly diagnosed cancer; (2) chemotherapy or radiotherapy in the past two years; (3) incomplete chemotherapy or delay in the first course of chemotherapy (>7 days after PCS); (4) simultaneous use of other antineoplastic agents, antiangiogenic agents, or targeted therapy	55 months	Paclitaxel was administered at a dose of 80 mg/m2 plus carboplatin (AUC 5) was administered triweekly	Paclitaxel was administered at a dose of 80 mg/m2 plus cisplatin was administered triweekly at a dose of 20 mg/m2
3	Neijt et al. [21]	2000	208	(1) Histologically verified epithelial ovarian carcinoma (based on the 1973 WHO histologic classification); (2) FIGO stages IIB to IV.	(1) WHO performance status of 4; (2) patients under 18 and older than 75 years of age; (3) complete bowel obstruction or the presence of symptomatic brain metastases; (4) previous chemotherapy or radiotherapy; (5) inadequate bone marrow function (WBC count 3.0 * 109 /L or platelet count 100 * 109 /L); (6) inadequate renal function (serum creatinine level >120 µmol/L or creatinine clearance 60 mL/min/1.73 m2), and liver function (bilirubin level 25 mmol/L); ( 6) History of documented myocardial infarction within 6 months prior to randomization; (9) patients having borderline tumors, abdominal adenocarcinoma of unknown origin or second malignant disease; (9) incomplete follow-up, an active infection, or other serious medical conditions impairing the ability of the patient to receive protocol treatment, including prior allergic reactions to drugs that contained Cremophor EL.	37 months	The paclitaxel dose of 175 mg/m2 was administered as a 3-hour infusion followed by a carboplatin dose calculated according to a target area under the plasma concentration- time curve of AUC 5 by the following formula: dose (mg)= 5* (glomerular filtration rate + 25).	Paclitaxel dose of 175 mg/m2 was administered as a 3-hour infusion followed by Cisplatin at a dose of 75 mg/m2.
4	Ozols et al. [19]	2003	720	(1) Women with pathologically verified stage III epithelial ovarian cancer who underwent a staging laparotomy with cytoreduction and those with residual disease less than or equal to 1.0 cm in diameter; (2) GOG performance status of 0 to 2, WBC Count at least 3,000/L, platelets at least 100,000/ L, serum creatinine 2.0 mg/dL or less and serum bilirubin and aspartate aminotransferase values of no more than 2.	(1) Previous chemotherapy; (2) borderline tumors.	36 months	Carboplatin was administered at an AUC of (7.5mg/mL/min) and paclitaxel (175 mg/m2) was administered as a 3-hour infusion over six courses every 3 weeks.	Cisplatin (75 mg/m2) was administered intravenously at 1 mg/min and Paclitaxel (135mg/m2) intravenously as a 24-hour continuous infusion every 3 weeks for a total of six courses.
5	Griemel et al. [22]	2006	783	(1) Histologically verified FIGO stages IIB-IV of ovarian cancer.	(1) Incomplete toxicity data; (2) more than 50% missed response.	6 months	Paclitaxel (185mg/m2) was administered intravenously for 3 hours and carboplatin was administered intravenously for 30 to 60 minutes.	Paclitaxel (185mg/m) was administered intravenously for 3 hours plus cisplatin (75 mg/m2) was administered intravenously over 6 courses every 3 weeks.

**Table 3:** Baseline characteristics of the included patients in this meta-analysis PT: paclitaxel-cisplatin; TC: paclitaxel-carboplatin; N/A: not applicable; U/K, unknown; ECOG: Eastern Cooperative Oncology Group

Name of author and year	No of patients		Mean Age		Grading of Tumor		Staging of Tumor		No. of Treatment Cycles		ECOG	
	PT	TC	PT	CT	PT	TC	PT	TC	PT	TC	PT	TC
Bois et al. [18]	386	397	N/A	N/A	U/K=33, 1=61, 2=284, 3=405.	U/K=20, 1=36, 2=145, 3=96.	<3=29, 3=295, 4=62.	<3=37, 3=268, 4=72.	0=2, 1-5=60, 6=306, >6=18.	0=2, 1-5=47, 6=323, >6=25.	0=191, 1=159, 2=136, 3=N/A.	0=198, 1=166, 2=33, 3=N/A.
Huang et al. [20]	18	22	59.4 (9.4)	58.4 (9.4)	U/K=N/A, 1=N/A, 2=N/A, 3=18.	U/K=N/A, 1=N/A, 2=N/A, 3=22.	<3=N/A, 3=18, 4=N/A.	<3=N/A, 3=22, 4=N/A.	0=N/A, 1-5=N/A, 6=18, >6=N/A.	0=N/A 1-5=N/A, 6=22, >6=N/A.	0=17, 1=N/A, 2=1, 3=N/A.	0=22, 1=N/A, 2=1, 3=N/A.
Neijt et al. [21]	108	100	N/A	N/A	U/K=25, 1=5, 2=36, 3=42.	U/K=22, 1=11, 2=20, 3=47.	<3=11, 3=75, 4=22.	<3=11, 3=70, 4=19.	0=N/A, 1-5=497, 6=85, >6=84.	0=N/A 1-5=481, 6=89, >6=130.	0=66, 1=37, 2=3, 3=2.	0=53, 1=53, 2=8, 3=6.
Ozols et al. [19]	400	392	N/A	N/A	U/K=N/A, 1=44, 2=139, 3=217.	U/K=N/A, 1=35, 2=141, 3=216.	N/A	N/A	0=N/A, 1-5=57, 6=341, >6=N/A.	0=N/A, 1-5=49, 6=342, >6=N/A.	0=N/A, 1=44, 2=139, 3=217.	0=N/A, 1=35, 2=141, 3=216.
Griemel et al. (2006) [22]	197	219	57.0 (9.24)	56.6 (10.72)	N/A	N/A	<3=117, 3=N/A, 4=80.	<3=115, 3=N/A, 4=104.	N/A	N/A	N/A	N/A

Risk of Bias Assessment and Publication Bias

**Figure 2:**
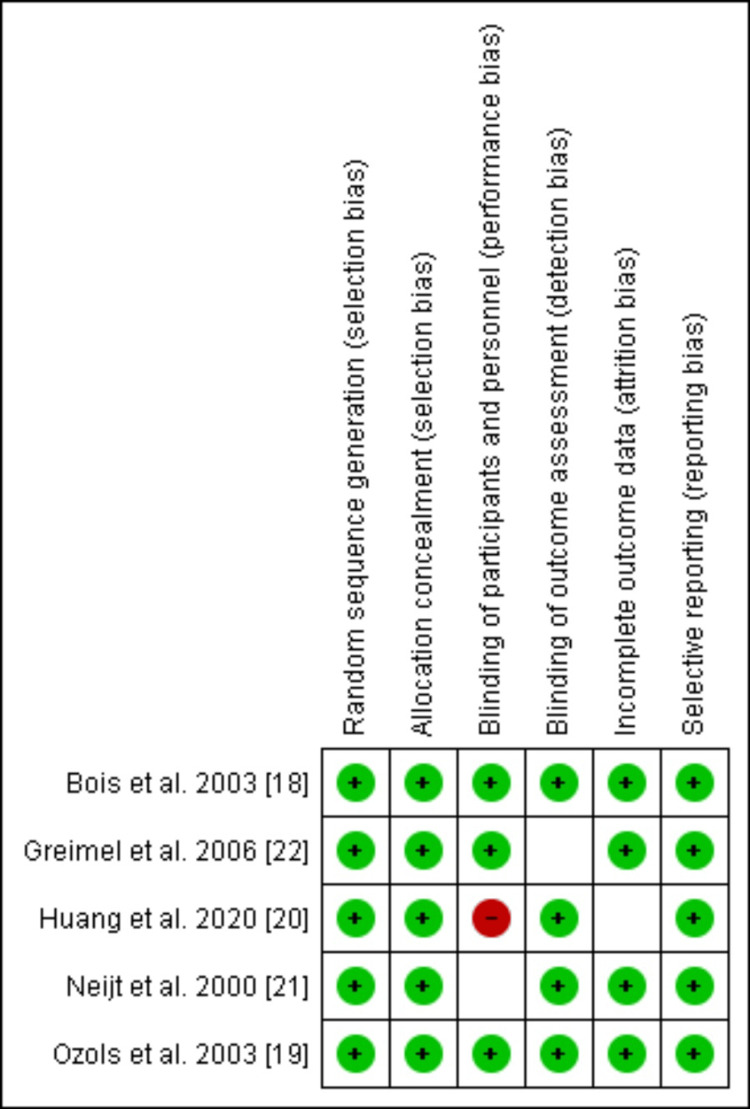
Risk of bias figure showing the risks of the included randomized controlled trials (RCTs)

**Figure 3:**
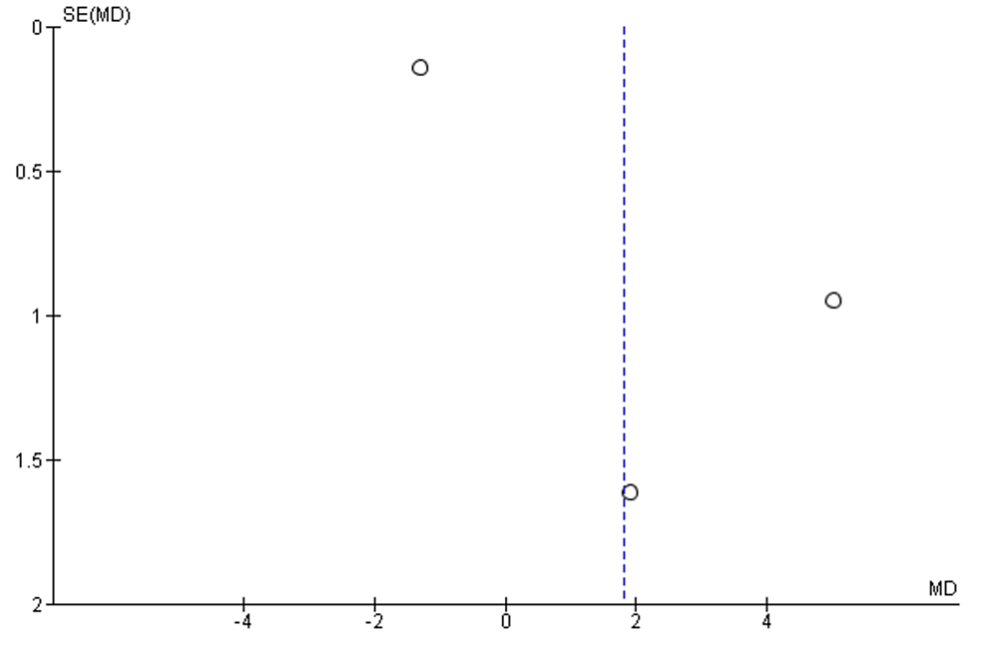
Funnel plot of progression-free survival

**Figure 4:**
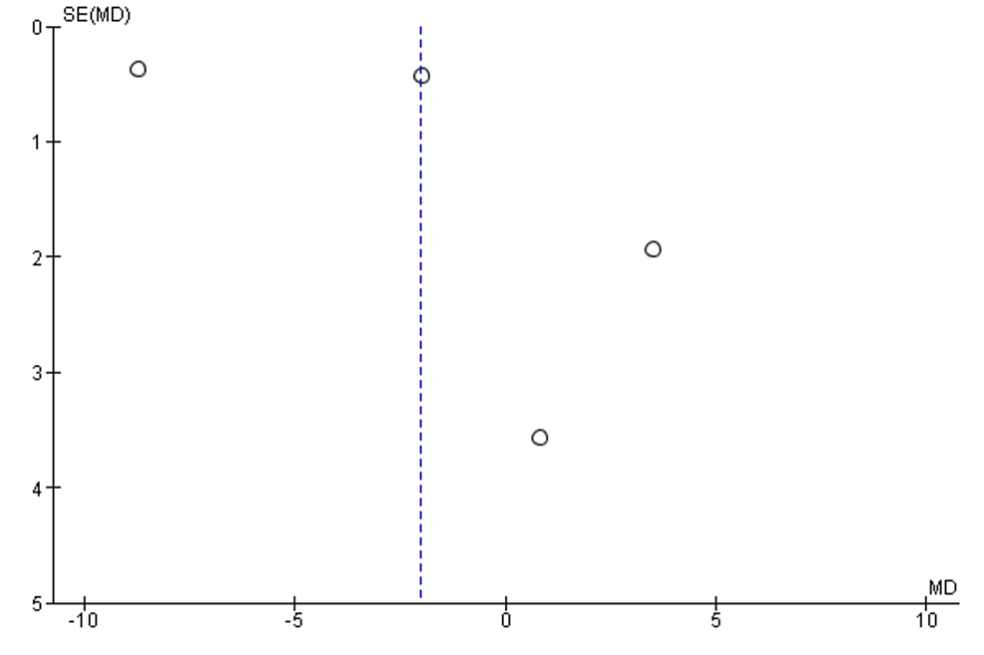
Funnel plot of overall survival

**Figure 5:**
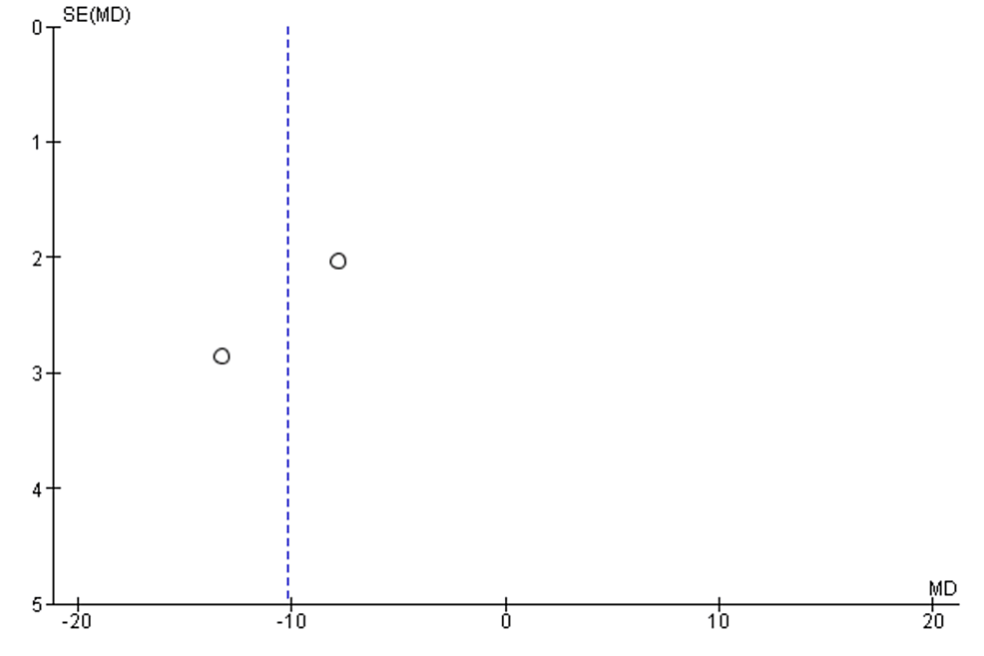
Funnel plot of quality of life

**Figure 6:**
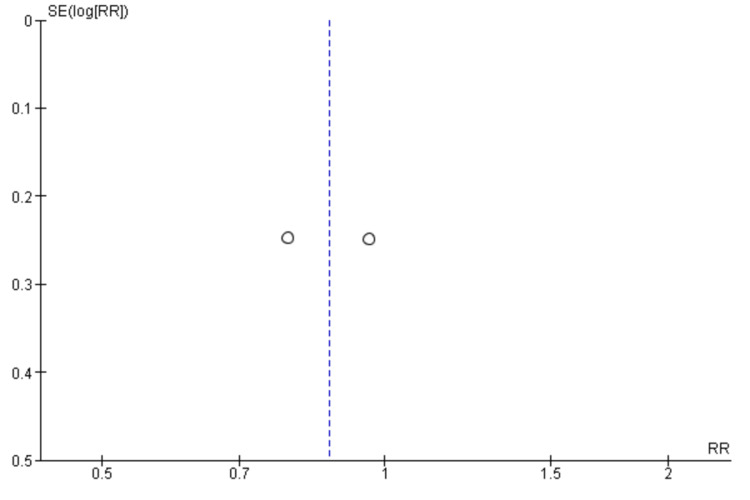
Funnel plot of complete response rate

**Figure 7:**
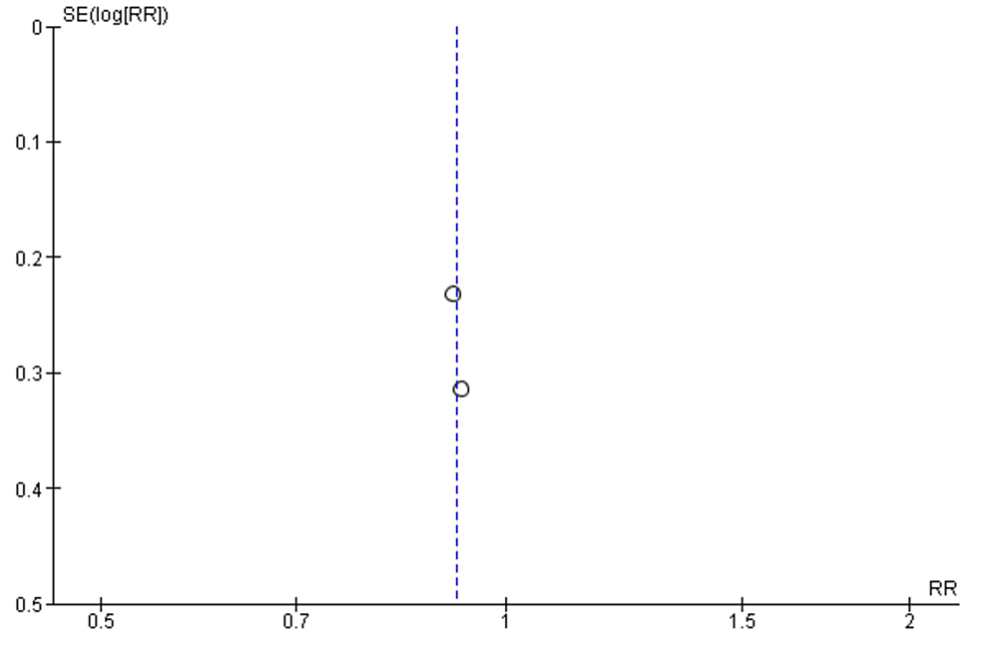
Funnel plot of partial response rate

Two researchers independently performed the risk of bias for all the included studies. Two out of the five RCTs showed a low risk of bias in all domains [18, 19]. One study showed a high risk of bias in one domain i.e. in the domain of performance bias as well as an unclear risk in another domain i.e. attrition bias [20]. Two studies showed unclear bias in two separate domains (detection bias and performance bias, respectively) [21,22] (Figure 2). The funnel plots were made to assess the publication bias. According to the plots, the outcomes remained impervious to any potential bias (Figures 3-7).

Primary Outcomes

**Figure 8:**
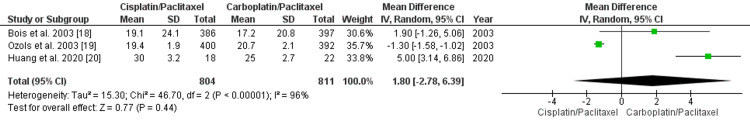
Forest plot of progression-free survival SD: standard deviation; CI: confidence interval

Progression-free survival time (PFS): PFS time was reported in three out of five studies included in the meta-analysis [18-20]. Patients in the cisplatin/paclitaxel group were noticed to have a higher progression-free survival time as compared to the patients taking the carboplatin/paclitaxel regimen (WMD = 1.80; 95% CI -2.78, 6.39; p-value = 0.44; I2 = 96%). High in-study heterogeneity was observed as shown in Figure 8.

**Figure 9:**
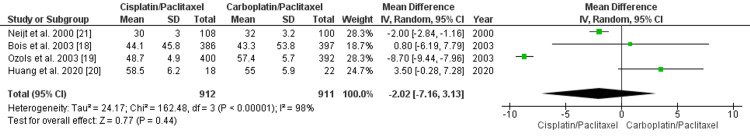
Forest plot of overall survival SD: standard deviation; CI: confidence interval

OS: OS was found to have an association with four out of five studies [18- 21]. The pooled analysis assessed an insignificant decrease in OS in the cisplatin/paclitaxel group relative to carboplatin/paclitaxel (WMD = -2.02; 95% CI -7.16, 3.13; p-value = 0.44; I2 = 98%). High in-study heterogeneity was observed as shown in Figure 9.

**Figure 10:**
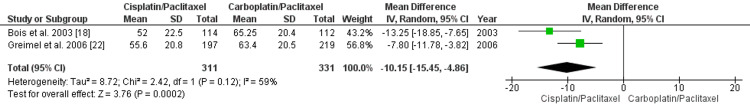
Forest plot of quality of life SD: standard deviation; CI: confidence interval

Quality of life (QoL): Two out of five studies included in the meta-analysis reported Quality of Life [18,22]. On analysis, there was a significant decrease in QoL in the cisplatin/paclitaxel group relative to carboplatin/paclitaxel (WMD = -10.15; 95% CI -15.45, -4.86; p-value = 0.0002; I2= 59%). Moderately low in-study heterogeneity was observed as shown in Figure 10.

**Figure 11:**
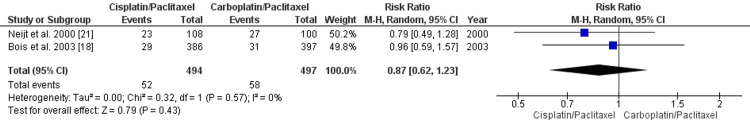
Forest plot of complete response rate CI: confidence interval

Complete clinical response (CRR): CRR was reported by two studies in the meta-analysis [18, 21]. Statistical analysis revealed an insignificant decrease in the CRR in the cisplatin/paclitaxel group as opposed to carboplatin/paclitaxel (RR = 0.87; 95% CI 0.62, 1.23; p-value = 0.43; I2 = 0%). No in-study heterogeneity was observed as shown in Figure 11. 

**Figure 12:**
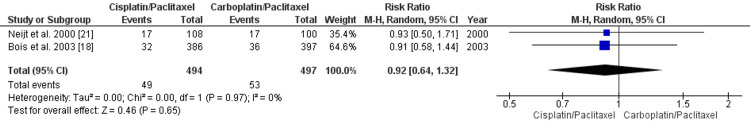
Forest plot of partial response rate CI: confidence interval

Partial response rate (PRR): PRR was reported by two of the five studies in the meta-analysis [18, 21]. A combined statistical analysis demonstrated a non-significant decrease observed in the PRR for patients taking cisplatin/paclitaxel as opposed to those using carboplatin/paclitaxel (RR = 0.92; 95% CI 0.64, 1.32; p-value = 0.65; I2 = 0%). No in-study heterogeneity was observed as shown in Figure 12.

Secondary Outcomes

Anemia was reported by four out of five studies [18,20-22]. An analytic assessment revealed a non-significant reduced risk of anemia in the cisplatin/paclitaxel group compared to the carboplatin/paclitaxel group (RR = 0.65; 95% CI 0.25, 1.52; p-value = 0.32; I2 = 37%). Moderately low in-study heterogeneity was observed. Thrombocytopenia was reported by five studies [18-22]. Statistical analysis indicated a significant decrease in the risk of thrombocytopenia in cisplatin/paclitaxel compared to carboplatin/paclitaxel (RR = 0.12; 95% CI 0.08, 0.18; p-value < 0.00001; I2 = 0%). No in-study heterogeneity was observed. Neutropenia was reported in five studies in both combinations given [18-22]. The pooled analysis exhibited a non-significant reduction in the risk of neutropenia in the cisplatin/paclitaxel group relative to carboplatin/paclitaxel (RR = 0.63; 95% CI 0.37, 1.06; p-value = 0.08; I2 = 96%). High in-study heterogeneity was observed. Leukopenia was reported by three studies [18, 19, 22]. Statistical analysis demonstrated a non-significant reduction in the risk of leukopenia in the cisplatin/paclitaxel drug group compared to carboplatin/paclitaxel (RR = 0.47; 95% CI 0.16, 1.39; p-value = 0.17; I2 = 97%). High in-study heterogeneity was observed. Neurological toxicity was reported by five studies [18-22]. Analytical assessment displayed a significant increase in the risk of neurological toxicity in the cisplatin/paclitaxel group relative to carboplatin/paclitaxel (RR = 1.58; 95% CI 1.17, 2.13; p-value = 0.003; I2 = 39%). Moderately low in-study heterogeneity was observed. Nausea/Vomiting was reported by four studies [18, 20-22]. According to the quantitative analysis, there was a significant elevation in the risk of nausea/vomiting in the cisplatin/paclitaxel group in comparison to carboplatin/paclitaxel (RR = 5.31; 95% CI 2.67, 10.54; p-value = < 0.00001; I2 = 83%). High in-study heterogeneity was observed. Myalgia/arthralgia was reported by three studies as a significant side effect of the drugs [18-19, 22]. Pooled analysis displayed a significant reduction in the risk of myalgia/arthralgia for cisplatin/paclitaxel relative to carboplatin/paclitaxel (RR = 0.69; 95% CI 0.52, 0.93; p-value 0.02; I2 = 0%). No in-study heterogeneity was observed. Nephrotoxicity came out to be an adverse effect in two studies [18, 22], the quantitative analysis suggested a non-significant increase in the risk of nephrotoxicity in the cisplatin/paclitaxel group (RR = 5.83; 95% CI 0.68, 50.27; p-value 0.11; I2 = 0%). Two studies came up with results displaying mucositis as an adverse effect, quantitative analysis of the findings showed a non-significant increase in the risk of mucositis in those taking cisplatin/paclitaxel [18,22] (RR = 1.09; 95% CI 0.26, 4.55; p-value 0.91; I2 = 0%). Two reports exhibited ototoxicity as a side effect; pooled analysis demonstrated a significant increase in the risk of ototoxicity in the cisplatin/paclitaxel group compared to the carboplatin/paclitaxel group [18,22] (RR = 3.13; 95% CI 1.01, 9.73; p-value 0.05; I2 = 0%). Hypersensitivity emerged as a side effect in two of the studies under consideration, quantitative analysis revealed a non-significant decrease in the risk for hypersensitivity in the cisplatin/paclitaxel group [18,22] (RR = 0.95; 95% CI 0.47, 1.95; p-value 0.90; I2 = 0%). Febrile neutropenia manifested itself as an adverse effect in two of the selected studies [18, 22]; statistically, a significant reduction was observed in the risk for febrile neutropenia in those taking cisplatin/paclitaxel (RR = 0.45; 95% CI 0.25, 0.83; p-value 0.01; I2 = 0%). Two of the studies revealed constipation as a side effect; statistical analysis displayed a non-significant increase in the risk for constipation in the cisplatin/paclitaxel group [18,22] (RR = 1.16; 95% CI 0.79, 1.69; p-value 0.45; I2 = 47%). Cardiotoxicity came out to be an adverse effect among two of the studies [18, 22]; quantitative analysis of the findings illustrated a non-significant increase in cardiotoxic events in patients receiving cisplatin/paclitaxel (RR = 1.28; 95% CI 0.68, 2.40; p-value 0.44; I2 = 0%). Dyspnea was exhibited as a side effect in two studies [18, 22]; quantitative analysis indicated a non-significant reduction in the risk of dyspnea in the cisplatin/paclitaxel group compared to the carboplatin/paclitaxel group (RR = 0.65; 95% C1 0.41, 1.03; p-value 0.07; I2 = 0%). Diarrhoea was reported as a side effect in two studies [18, 22]; quantitative analysis displayed a non-significant increase in risk for diarrhea in the group receiving cisplatin/paclitaxel (RR = 1.11; 95 % CI 0.57, 2.13; p-value 0.76; I2 = 0%). Edema was a side effect in two out of five studies [18, 22]; statistical analysis revealed a non-significant reduction in the risk for edema in the cisplatin/paclitaxel drug regime (RR = 0.34; 95% CI 0.02, 6.40; p-value 0.47; I2 = 70%) as shown in Table 4.

**Table 4:** Summary of forest plots of the secondary outcomes

Serial No.	Outcomes	Risk Ratio	95% Confidence Interval	p-value	Heterogeneity
1	Anemia	0.65	0.25-1.52	0.32	37%
2	Thrombocytopenia	0.12	0.08-0.18	<0.00001	0%
3	Neutropenia	0.63	0.37-1.06	0.08	96%
4	Leukopenia	0.47	0.16-1.39	0.17	97%
5	Neurological toxicity	1.58	1.17-2.13	0.003	39%
6	Nausea/Vomiting	5.31	2.67-10.54	<0.00001	83%
7	Myalgia/Arthralgia	0.69	0.52-0.93	0.02	0%
8	Nephrotoxicity	5.83	0.68-50.27	0.11	0%
9	Mucositis	1.09	0.26-4.55	0.91	0%
10	Ototoxicity	3.13	1.01-9.73	0.05	0%
11	Hypersensitivity	0.95	0.47-1.95	0.90	0%
12	Febrile neutropenia	0.45	0.25-0.83	0.010	0%
13	Constipation	1.16	0.79-1.69	0.45	47%
14	Cardiotoxicity	1.28	0.68-2.40	0.44	0%
15	Dyspnea	0.65	0.41-1.03	0.07	0%
16	Diarrhea	1.11	0.57-2.13	0.76	0%
17	Edema	0.34	0.02-6.40	0.47	70%

Drug Regimen Dosages

Different treatment doses of carboplatin/paclitaxel (TC) and cisplatin/paclitaxel (PT) were reported in the included studies. 

Bois et al. reported 185 mg/m2 of paclitaxel administered intravenously over three hours followed by carboplatin (AUC 6) administered intravenously over 30 to 60 minutes [18]. For the PT arm, they delivered the same dosage of paclitaxel as the TC arm in addition to the 75 mg/m2 of cisplatin intravenously over 30 minutes. 

The study by Ozols et al. was the only RCT in which paclitaxel was administered in different dosages in both arms [19]. In the TC arm, 175 mg/m2 of paclitaxel was given as a three-hour infusion over six courses every three weeks with carboplatin at an AUC of 7.5 mg/mL/min. In the PT arm, 135 mg/m2 of paclitaxel was administered intravenously as a 24-hour continuous infusion every three weeks for a total of six courses along with 75 mg/m2 of cisplatin given intravenously at a rate of 1 mg/min.

For the TC arm, Huang et al. reported 80 mg/m2 of paclitaxel with triweekly carboplatin (AUC 5) whereas for the PT arm, it was 80 mg/m2 of paclitaxel with triweekly 20 mg/m2 of cisplatin [20].

In one study, for both the TC and PT arm paclitaxel was administered at 175 mg/m2 as a three-hour infusion [21]. In the TC arm, carboplatin was given according to the area under the plasma-concentration time curve which was 5. In the PT arm, cisplatin was given at a dose of 75 mg/m2.

Griemel et al. reported using 185 mg/m2 of paclitaxel in both the TC and PT groups. The drug was administered intravenously over three hours [22]. In the TC arm, carboplatin dosage was calculated by milligrams area under the curve (GFR25) and administered intravenously over 30 to 60 minutes. In the PT arm, 75 mg/m2 of cisplatin was given through IV over six courses every three weeks. 

Discussion

In 2020, ovarian carcinoma was ranked as the third most prevalent cancer globally [23]. Considering the aggressively malignant nature of ovarian tumors [24], the chemotherapeutic management of advanced carcinomas after radical debulking surgery consisted of treatment with a taxane/platinum doublet [25]. For years, multiple researchers set out to answer one simple question: between carboplatin and cisplatin, which of the two was more efficacious and well-tolerated in terms of overall survival and quality of life when combined with paclitaxel? Our meta-analysis compiled findings from five RCTs (2239 participants) to provide quantitative results regarding the safety and efficacy profile of carboplatin/paclitaxel against cisplatin/paclitaxel. 

Among the primary outcomes, our meta-analysis demonstrated significant results for quality of life, thrombocytopenia, neurological toxicities, nausea/vomiting, and arthralgia/myalgias. Conventionally, biomedical markers such as progression-free survival were employed as endpoint assessments, with outcomes like quality of life often ignored [26]. While both progression-free and overall survival time are implicated as primary endpoints to evaluate treatment outcomes for any disease, neither has been established as the superior one. However, as post-progression therapy bears a confounding effect on overall survival time, researchers prefer to use progression-free survival time instead [27]. Moreover, research regarding the substitution of overall survival time with progression-free survival time has yielded insignificant results and therefore, both are used as measures for endpoint assessments [27,28].

While our meta-analysis revealed statistically insignificant results for progression-free survival time, overall survival, clinical response rate, and partial response rate, the statistically significant findings for quality of life serve as pivotal as they exhibited better outcomes for those taking the carboplatin/paclitaxel combination. Where most attempts at defining quality of life have been vague, health-related quality of life is currently described as a measure to evaluate the effect of disease on a person’s life including any impact of received treatment on routine functioning [26]. The quality of life deteriorates in patients with advanced ovarian cancer as depicted in a study that stated that these patients present with poor social and functional quality of life [29]. Therefore, the results of our meta-analysis for quality of life are of great clinical importance, especially bearing in mind the scarcity of previously available literature regarding the profound effects of this parameter on patients of ovarian carcinoma [30,31].

Seven toxicities were included in this meta-analysis and four yielded statistically significant findings i.e. thrombocytopenia, neurological toxicities, nausea/vomiting, and arthralgia/myalgias. Previous studies have demonstrated carboplatin’s dose-limited thrombocytopenic adverse effects and our findings were consistent with these results; consequently, the cisplatin/paclitaxel combination was more well-tolerated in terms of thrombocytopenia [13,32]. Additionally, patients taking the cisplatin-based combination had fewer complaints regarding arthralgia/myalgia when compared with carboplatin/paclitaxel. However, for neurological toxicities and nausea/vomiting, the carboplatin-based drug regime exhibited more favorable results. In the included secondary outcomes, only febrile neutropenia and ototoxicity yielded significant findings. Carboplatin exhibited fewer ototoxic side effects, while cisplatin showed a lower incidence of febrile neutropenia. Hitherto, carboplatin not only induces less neurotoxicity, nephrotoxicity, and ototoxicity compared to cisplatin, as indicated in a review [33], but it also presents a reduced risk of delayed nausea, as substantiated by a meta-analysis that compared carboplatin plus pemetrexed with carboplatin-paclitaxel [34]. Furthermore, other hematological side effects such as anemia, neutropenia, and leukopenia yielded inconclusive results. Leukopenia has been established as a prognostic indicator in patients undergoing carboplatin/paclitaxel treatment [35]. While our meta-analysis suggested a reduced risk of developing leukopenia, the results did not reach statistical significance. Conversely, neutropenia was not found to be a significant prognostic factor for patients taking carboplatin [35], and the pooled analysis indicated no significant difference in the risk of neutropenia between those taking cisplatin and carboplatin. A study reported that 30% of individuals receiving carboplatin for advanced ovarian cancer experienced anemia as an adverse effect [36]. However, the pooled analysis for anemia did not find significant differences between the two drugs, although cisplatin showed a slight advantage.

Significant heterogeneity was observed among the studies, with an I2 value exceeding 75% [17], for outcomes such as progression-free survival time, overall survival, leukopenia, neutropenia, and nausea/vomiting. A moderately low level of in-study heterogeneity, I2 values between 50 to 75% [17], was noted for quality of life and edema whereas a low level of in-study heterogeneity was observed for the remaining outcomes. These variations in heterogeneity were primarily attributed to several factors, including differences in patient age across the enrolled studies, variations in the follow-up duration, and variations in drug dosages. As explained in a study [37], these factors contributed to the observed heterogeneity. To address such high heterogeneities, we conducted a leave-one-out sensitivity analysis. This involved systematically excluding one study at a time and assessing its impact on heterogeneity for specific outcomes. For example, in our efforts to reduce heterogeneity in progression-free survival time, overall survival, neutropenia, leukopenia, and nausea/vomiting, we removed the study conducted by Ozols et al. [19]. After its exclusion, heterogeneity decreased significantly, specifically for progression-free survival time and neutropenia, the I2 values for both outcomes dropped from 96% to 64% and 20%, respectively (as shown in Figures 25 and 27). Similarly, for overall survival, heterogeneity dropped from 98% to 76%, and for leukopenia, it reduced from 97% to 0%. Lastly, heterogeneity in nausea/vomiting decreased from 83% to 0%.

Several outcomes were not included in this meta-analysis as they were reported in only one or two of the included studies and hence, they were not the primary focus of our research. First and foremost, only one out of the five selected studies included the outcomes of granulocytopenia and fever [21]. As such, they were not separately discussed in this analysis. Granulocytopenia, analogous to neutropenia, has been chronicled as one of the prevailing side effects of paclitaxel-induced chemotherapy, but it's often managed using granulocyte colony-stimulating factors, as indicated by a case report [38]. Despite the exclusion of fever from our research, it's worth emphasizing that fever during chemotherapy requires urgent medical attention. Physicians should avoid unnecessary treatments like prescribing antibiotics and conducting examinations after being informed about chemotherapy-associated fever on post-treatment days 3 and 4 [39]. Alopecia as an outcome was not highlighted in this analysis due to its inclusion in only one study [18], alopecia is a notable side effect of chemotherapy and it has profound implications on a patient’s body image and self-concept [40].

Myelosuppression, defined as reduced bone marrow activity that culminates in decreased red cell count, white cell count, and platelet count, was not included as an outcome separately as it was adequately explained through outcomes such as anemia, leukopenia, and thrombocytopenia, respectively. Myelosuppression in chemotherapy is often associated with older age, as indicated in the clinical trial conducted by Greimel et al [22]. Fatigue, an outcome mentioned only in Greimel et al. [22], is a significant predictor for quality of life as a substantial number of ovarian cancer patients experience fatigue [41]. Appetite loss, another mediator for quality of life, was reported by only one study [22] and was not included in our meta-analysis despite results from a study indicating how appetite loss impairs life for patients with cancer recurrence [42]. While insomnia was not included among outcomes for this study as only one study reported it [22], it has been established as a major factor contributing to a patient’s assessment of quality of life [43]. Again, severe hemorrhage and mortality were excluded from our study on the grounds that they were reported by only one study [22]. Lastly, our meta-analysis did not focus on the inclusion of any metabolic toxicities. Outcomes such as hypomagnesemia and abnormal electrolyte levels were reported in only one study [19]. Hypomagnesemia can result from cardiovascular medications used to treat cardiovascular disorders and from kidney-related complications of cancer and chemotherapy [44], while electrolyte imbalances (e.g., sodium, potassium, calcium, and phosphate) are commonly encountered during platinum-based chemotherapy [45]. These outcomes were excluded due to their limited representation in the available studies and their limited relevance to the primary objectives of our study.

There were several limitations to this pooled analysis. Firstly, our meta-analysis was restricted to only a few countries such as Europe, Germany, Taiwan, America, and Europe; the findings might not be completely relevant to patients from other locations. Along with this, the dosages administered to patients were different among all five studies. Moreover, the studies adhered to different grading systems for the included toxicities which can lead to differences in results across all studies. Secondly, only RCTs were included in this meta-analysis and all articles not published in English were excluded. This might have excluded some of the important differences observed between the studies. Furthermore, a possible limitation of this study can be the use of a generic QoL measure without a cancer-specific instrument by Greimel et al. [22]. The sensitivity to treatment-related changes may have arisen due to a cancer location-specific measure. Additionally, the EORTC ovarian cancer module was not fully functional and was not developed when patients were included in this study. This study also had the limitation of returning incomplete QoL forms or patients who dropped out of the trial and did not undertake the QoL assessment. The trial conducted by Huang et al. included the retrospective collection of data in the environment [20]. In addition, there was a high risk of selection bias in this study as it only comprised patients who underwent PCS with a surgical affirmation of FIGO IIIC serous-type ETOC and PPSC, and undertook six cycles of shorter time intervals between treatments with paclitaxel-platinum compounds. 

## Conclusions

Based on the evidence we have gathered, the carboplatin/paclitaxel combination appears to offer a better QoL for ovarian cancer patients compared to cisplatin/paclitaxel. However, it is important to note that one significant drawback of carboplatin/paclitaxel is its potential for myelosuppression, particularly thrombocytopenia. On the positive side, this combination tends to have lower gastrointestinal and neurological toxicity than cisplatin/paclitaxel, reinforcing its superiority in terms of patient well-being. Nevertheless, it’s worth mentioning that patients receiving carboplatin/paclitaxel may experience increased muscle and joint pain.

We are inclined to believe that the carboplatin/paclitaxel combination should be given precedence over cisplatin/paclitaxel owing to the relatively better quality of life it affords to patients, provided appropriate clinical measures are taken to manage its myelosuppressive effects. It’s important to acknowledge that most of the literature on this topic is dated, and more recent trials are needed to reaffirm these findings in the context of current medical practice.
